# Routine Cord Blood Platelet Counts and Potential for Severe Neonatal Alloimmune Thrombocytopaenia (NAIT): A Cohort Study of 12 Yr. Experience at Middlemore Hospital, New Zealand

**DOI:** 10.1111/ajo.70065

**Published:** 2025-11-21

**Authors:** Galama Vela, Jill H. Meyer, Michael P. Meyer

**Affiliations:** ^1^ Medical Laboratory Sciences, Medicine and Health Sciences University of Papua New Guinea Oceania Papua New Guinea; ^2^ Department of Biomedicine and Medical Diagnostics Auckland University of Technology Auckland New Zealand; ^3^ Neonatal Unit, KidzFirst Middlemore Hospital Auckland New Zealand; ^4^ Department of Paediatrics Child and Youth Health University of Auckland Auckland New Zealand

**Keywords:** cord blood, neonatal alloimmune thrombocytopaenia (NAIT), newborn infant, platelet antigens, thrombocytopaenia

## Abstract

**Background:**

Neonatal alloimmune thrombocytopaenia (NAIT) is a rare but potentially serious condition where maternal antibodies result in destruction of foetal and neonatal platelets. At Middlemore Hospital in south Auckland, routine cord blood platelet counts were performed over many years.

**Aims:**

These were twofold: To determine the prevalence of severe thrombocytopaenia (TP) and severe NAIT and investigate platelet counts in siblings of infants with TP.

**Materials and Methods:**

Cord blood was collected on all hospital births over 500 g over a 12‐year period (2005–2016) and term infants with TP (< 150 × 10^9^/L) selected. Records of infants with severe TP (< 50 × 10^9^/L) were reviewed for potential NAIT cases. Records of siblings of infants with any degree of TP were also reviewed to examine the potential for NAIT in affected families.

**Results:**

Of 68910 births, 62083 platelet counts were suitable for analysis and 641 term infants had TP (1%) with 16 having severe TP (0.025%). NAIT or potential NAIT was judged clinically to be present in half of these (0.013%). Most cases were of European ethnicity with a Maori infant and a Tongan infant also being possible cases. No serious complications were identified. 5% of siblings of infants with TP had low counts with only one infant having a likely diagnosis of severe NAIT.

**Conclusions:**

Severe TP was uncommon amongst infants born in south Auckland where mothers of Maori and Pacific Island ethnicity make up the majority of the population. Performance of routine cord platelet counts was of limited value in detecting potential cases of severe NAIT.

## 1 Introduction

Neonatal alloimmune thrombocytopaenia (NAIT) is analogous to haemolytic disease of the fetus and newborn (HDFN) most commonly associated with Rhesus or ABO incompatibility. The TP seen in NAIT (typically below 50 × 10^9^/L) [1] is due to the destruction of foetal platelets by maternal allo‐antibodies against paternally acquired antigens on the foetal platelets [2]. Risks to the fetus or newborn range from incidental TP through a purpuric rash to intracranial haemorrhage with neurologic sequelae or even death [3]. Firstborn infants with NAIT can be affected [4]. Subsequent siblings are also at risk, thus identification of NAIT is important for all subsequent pregnancies in that family [5].

The incidence amongst European women is about 1 in 1000–1 in 2000 [6], but in New Zealand, and particularly south Auckland with a predominant demographic of Maori and Pacific Island peoples, the incidence is unknown. There is no routine screening for NAIT at Middlemore Hospital. However, routine performance of cord full blood counts was standard practice at Middlemore Hospital from 2005–mid‐2016. Our aim was the identification of NAIT or suspected NAIT cases, and, if these were sufficiently common, this would provide reasons to continue cord blood testing. Because NAIT can affect multiple siblings in the same family, we sought also to review platelet counts in siblings of healthy term infants with NAIT or unexplained TP.

We considered a platelet count below 50 × 10^9^/L in a healthy newborn suggestive of severe NAIT (other potential causes being placental insufficiency or maternal idiopathic thrombocytopaenia) [1] and this would warrant NAIT‐specific analysis [7, 8, 9]. Although TP in cord blood samples at Middlemore Hospital was identified in both term and preterm infants, the focus of this report was on term infants (37–41 weeks). Two cohorts are described: potential severe NAIT cases and potential NAIT cases in siblings of term infants with TP.

**TABLE 1:** Term infants with severe thrombocytopaenia (less than 50 × 10^9^/L) identified from cord blood testing.

	Year	Ethnicity	Gest	Weight	Sex	PLT	Diagnosis	Other significant lab results	Outcome	Siblings
1	2008	E	39	3600	M	7	NAIT	HPA‐1 antibody	2 IG No PT	#2 Tab1 # 14 Tab2
2	2010	E	39	3350	M	5	NAIT	HPA‐1 antibody	2 PT 1 IG	#1 Tab1 #14 Tab2
3	2007	E	39	2700	M	27	NAIT	HPA‐1 antibody	No PT	nil
4	2008	E	37	2330	F	9	P NAIT Rash	NT	N 42d	4 sibs #15 Tab2
5	2008	M	38	3190	F	13	P NAIT	NT	No FU	3sibs N counts at birth
6	2005	E	38	3130	F	32	P NAIT	NT	N 21d VSD	No
7	2005	E	40	2710	F	42	P NAIT	NT	No FU	No
8	2014	T	39	3000	M	46	P NAIT	NT	No FU	No
9	2013	C	39	3300	M	32	ITP			No
10	2005	I	39	3565	M	41	ABO Kernicterus	SBR 630	N 18d CP, delay	No
11	2006	S	38	2465	M	35	Bacteraemia	E Coli	Died	No
12	2005	I	37	2100	M	41	Pseudo TORCH	PT antibody negative	Died age 3y	No
13	2006	S	38	2400	M	29	Severe IUGR	NT	Resolved	2003 ND
14	2012	T	38	5500	F	22	SB TIIDM, PET	NT	Poor control HT mec	#12 Tab2 2013 ND
15	2015	E	40	3835	M	35	SB	Acidosis cord blood	Perinatal hypoxia PM	2 sibs N
16	2012	S	37	3220	F	27	HDN	Anti E, anti c anti JKa	N 14d Club foot	2013 N

Abbreviations: ABO, ABO blood group incompatibility; CP, cerebral palsy; E, European; Ethnicity C, Cook Island Maori; Gest, Gestational age in weeks; HDFN, haemolytic disease of the fetus and newborn; HPA, human platelet antigen; HT, hypertension; I, Indian; IG, intravenous immunoglobulin; ITP, idiopathic thrombocytopaenic purpura; IUGR, intrauterine growth restriction; M, NZ Maori; mec, meconium exposed; N, normal; NAIT, neonatal alloimmune thrombocytopaenia; ND, not done; No FU, no follow up; NT, not tested; P NAIT, potential NAIT; PET, preeclamptic toxaemia; PLT, platelet count × 10^9^/L; PM, post mortem; PT, platelet transfusion; S, Samoan; SB, stillbirth; SBR, serum bilirubin; sibs, siblings; T, Tongan; Tab, Table; TIIDM, diabetes; TORCH, toxoplasmosis, other, rubella, cytomegalovirus, herpes; VSD, ventriculoseptal defect; Weight, birth weight (g); Year, Year of birth.

**TABLE 2:** Thrombocytopaenia in siblings of infants identified with low platelet counts from cord blood testing.

No.	Maternal details			First known TP				Subsequent cases		
	Ethnicity	Parity*	Complications	Child no	Yr	PT	Sex	Year	PT	Sex
1	Tongan	G1P1	Mec FD	1	2013	88	M	2014	135	F
2	Fijian	G7P6	GM ITP	6	2014	138	F	2018	38	M
3	Samoan	G3P3	TIIDM anti E	2	2006	105	M	2010 2012	109 130	M M
4	Samoan	G5P3	GM	3	2011	146	M	2013	132	F
5	CI Maori	G2P1	ITP	1	2005	34	M	2007	100	M
6	Tongan	G6P6	GM	6	2006	144	F	2008	133	F
7	Indian	G2P2		2	2003	139	F	2007	108	F
8	Tongan	G12 P4	GM	3	1998	16	F	2007 2009	129 134	M M
9	Indian	G1P1		1	2007	134	F	2013	92	F
10	Tongan	G14P12	ITP GM	12	2004	34	F	2007	136	F
11	Fijian	G4P3	GDM	3	2005	148	F	2007	100	F
12	Tongan	G3P3	SB, MAS GDM	3	2010	133	F	2012 #14 Tab1	22	F
13	Samoan	G3P3	TI DM	3	2009	127	F	2010	135	M
14	European	G2P3	NAIT Twins	1	2003 2003	40 15	F M	2008 2010 #1, #2 Tab1	< 10 5	M M
15	European	G3P3		2	2005	100		2008 #4 Tab1	9	F

Abbreviations: CI Maori, Cook Island Maori; Complications, complications in mother during pregnancy of child identified in survey years; F, female; FD, foetal distress; GDM, gestational diabetes mellitus; GM, grand multip; ITP, idiopathic thrombocytopaenic purpura; M, male; MAS, meconium aspiration syndrome; Mec, meconium exposed; NAIT; neonatal alloimmune thrombocytopaenia; PT, platelet count × 10^9^/L; SB, stillbirth; TI DM, type 1 diabetes mellitus; TIIDM, type 2 diabetes mellitus; Yr, year.

* Parity refers to that at delivery of child in survey years; not the first case in the family.

**TABLE 3:** Summary of population‐based studies reporting thrombocytopaenia and Neonatal Alloimmune Thrombocytopaenia at birth (including current south Auckland study).

Study	No. of samples	Source	TP	Severe TP	NAIT	Sample selection
France [10]	5632	Cord	0.9%	0.14%	0.15%	Unselected
Switzerland [11]	8388	Cord	0.9%^a^	0.12%	0.08%^b^	Unselected
Canada [12]	15932	Cord		0.12%	0.06%	Selected
Finland [13]	4489	Cord	2%	0.24%	0.15%	
Norway [8]	100488	Maternal blood		0.05%		HPA‐1a neg screening
Poland [14]	24101	Cord	0.5% (< 100)	0.15%	0.04%	Unselected
South Auckland	62083	Cord	1%	0.025%	0.013%^c^	Unselected
Systematic Review [15]	59425	Cord		0.15%	0.04% ICH0.01%	Literature review

Abbreviations: NAIT, neonatal alloimmune thrombocytopaenia; TP, thrombocytopaenia < 150 × 10^9^/L, severe TP < 50 × 10^9^/L.

^a^ Confirmed TP on infant blood test 0.5%.

^b^ Calculated from paper.

^c^ Confirmed or potential.

## 2 Materials and Methods

### 2.1 Routine Cord Blood Platelet Counts

Thrombocytopaenia (TP) was defined as a platelet count of < 150 × 10^9^/L and subdivided into severe (< 50 × 10^9^/L), moderate (50–< 100 × 10^9^/L) and mild (100–< 150 × 10^9^/L). Between 2005 and 2016 the haematology laboratory performed routine cord full blood counts, including platelet counts, on every infant born at Middlemore Hospital (including stillbirths and infants over 500 g). Midwifery staff performed venepuncture on the umbilical vein and utilised an EDTA tube (0.5 mL). Specimens were given a laboratory number and processed on a variety of haematology analysers (Beckamnn Coulter Gen S and Sysmex XE‐series). The reference range throughout the study period remained unchanged. Results were recorded in a laboratory database and electronic hospital record. Samples that did not meet standards for labelling or were clotted or had microclots were not routinely repeated, and, if not repeated, cases were excluded.

The cord blood platelet count was the focus of this report, unless the early postnatal sample (usually within 24 h) was done to confirm a potentially abnormal cord blood count result (e.g., because of microclots). This report considered only term infants (37–41 weeks). All preterm infants below 35 weeks were routinely admitted to neonatal care and, in general, had other conditions which could explain the low platelet counts, such as maternal pre‐eclampsia, or infection, and were not usually investigated for NAIT unless the TP was unexplained, moderate or severe, or prolonged (over 2 weeks).

### 2.2 Potential Severe NAIT Cases 2005–2016

#### 2.2.1 Inclusion

Term infant (37–41 weeks).

Severe TP < 50 × 10^9^/L at birth.

#### 2.2.2 Exclusion

Preterm < 37 weeks.

TP > 50–< 150 × 10^9^/L.

Once term infants with severe TP were identified in the laboratory database, the electronic medical report was reviewed.

#### 2.2.3 Review of Cord Blood Platelet Results

The primary caregiver for healthy term infants was usually a midwife (hospital or independent practitioner). Infants admitted to the neonatal unit (symptomatic term infants or preterm infants < 35 weeks' gestational age) were cared for by the neonatal team who were responsible for reviewing results.

### 2.3 Potential NAIT Cases in Siblings of TP Infants

Where thrombocytopenia of any severity occurred in an otherwise healthy infant, sibling electronic records and platelet counts were reviewed to determine if there were potential cases of NAIT in the family and investigate for adverse outcomes such as bleeding or intracerebral haemorrhage.

We reasoned that term infants born between 2005 and 2016 with mild or moderate TP and conditions which could explain the TP (e.g., infection) were unlikely to be cases of NAIT and no investigation of siblings was carried out in these symptomatic infants.

From the original laboratory database, we selected the following cases of TP:

#### 2.3.1 Inclusion

Term infant (37–41 weeks).

Healthy asymptomatic infant with unexplained TP of any severity.

Count < 50 × 10^9^/L at birth (whether symptomatic or not).

#### 2.3.2 Exclusion

Symptomatic term infant with other medical or surgical causes to explain the TP and platelet count > 50 × 10^9^/L.

We searched for siblings on a hospital database where children born to the same mother were recorded. Hospital birth electronic medical records (including cord platelet counts) for these siblings were reviewed.

Some of the siblings were born before the survey years (2005–16). If these siblings also had TP, we referred to these as the ‘initial’ case in the family. (We realised some of the siblings might have a different parent and might not be the first case).

Ethnicity was the prioritised self‐identified ethnicity reported by the mother and obtained from the electronic record.

No adjustments were made for missing data.

Statistics: We planned to calculate prevalence rates with 95% confidence intervals and Chi‐square tests and odds ratios with confidence intervals for comparisons.

Ethical approval for review of hospital records was obtained from the Middlemore Hospital Research Office and the Auckland University of Technology Ethics Committee (2017/323).

## 3 Results

**FIGURE 1:**
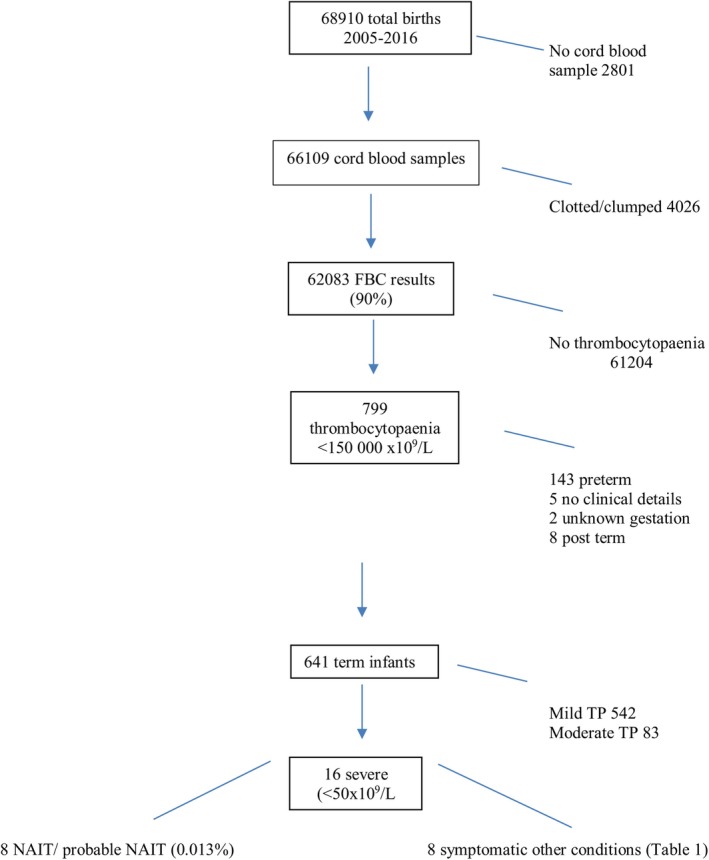
Flow diagram showing evaluation of cord blood platelet counts. FBC, full blood count; NAIT, neonatal alloimmune thrombocytopaenia; TP, thrombocytopaenia.

There were 68910 births during the period under review; 62083 platelet counts were suitable for analysis and 641 term infants had TP (Figure 1).

### 3.1 Severe Thrombocytopaenia (< 50 × 10^9^/L) Identified From Routine Cord Blood Platelet Counts

There were 16 cases with severe TP which were either confirmed low on repeat counts within 24 h or not repeated and assumed to be correct (Table 1).

### 3.2 NAIT or Potential NAIT (pNAIT)

As shown in Table 1, there were eight cases of confirmed or potential severe NAIT (0.013% of those with cord blood results). Cases #1 and 2 were European siblings born within the time frame under review. The initial case in this family was a twin with severe TP born before the study commenced. All affected children in this family had PLA‐1 antibodies. Case #1 had platelet transfusions and intravenous immunoglobulin and Case #2, intravenous immunoglobulin. There were no foetal losses in this family.

Case #3, born to European parents, had PLA‐1 antibodies detected. There were no other siblings or reported foetal losses.

Cases #4to #8 These five cases were not tested for platelet antibodies and three had no follow up platelet counts (cases#5, 7, 8); subsequent review of clinical records indicated that all three remained well. Case #5 had three siblings whose platelet counts were normal at birth. Cases #7 and 8 had no siblings. Two of the 5 pNAIT (#4, #6) were followed up until counts were normal. Case #4 had a sibling with a platelet count of 100 × 10^9^/L at birth, suggesting some risk in future pregnancies but the family was completed after the birth of this child. Overall, there were no clinical episodes of bleeding or death in the NAIT or pNAIT cases. Apart from case #4 who presented with a petechial rash, the other NAIT or pNAIT cases were asymptomatic.

**Other Causes** Eight of the 16 with severe TP had other causes to explain the low counts. There was one case each of ITP, severe IUGR, pseudotorch syndrome (with negative platelet antibodies) and E coli bacteraemia. There were two with HDFN. One of these was an ABO incompatibility (case #10) who required exchange transfusion and developed kernicterus. The other (#16) had multiple red cell antibodies. There were two stillbirths: case #15 had a post‐mortem with a final diagnosis of perinatal hypoxia and no evidence of bleeding. The other stillbirth (case #14) was born to a mother of Tongan ethnicity. She was a diabetic whose disease was poorly controlled and she also had hypertension and pre‐eclampsia. The stillborn infant did not have a post‐mortem although a physical examination did not reveal pallor or signs of bleeding. The cause of death was assigned to complications of the pregnancy. She had a child 2 years prior who had a platelet count of 133 × 10^9^/L (Table 2). Although difficult to exclude NAIT in this case, there were other more likely reasons for the stillbirth.

### 3.3 Potential for NAIT Amongst Siblings of Asymptomatic Thrombocytopaenic Newborns Identified by Cord Blood Results

**FIGURE 2:**
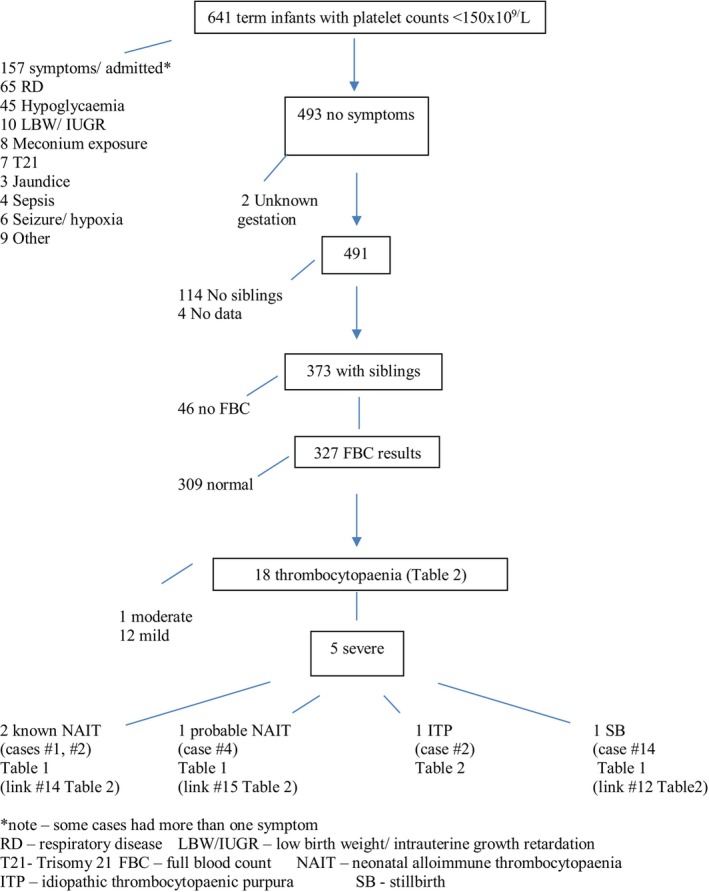
Flow diagram of platelet counts in siblings of term infants identified as having thrombocytopaenia.

Of the 373 asymptomatic term infants with siblings during the study period, 327 (88%) of the siblings had platelet counts recorded, and 18 (5%) of these had low counts (Figure 2). As noted in the methods section, some of these siblings were born before the current audit years. The first case of TP in each family was noted (Table 2). Overall, there were five cases of severe TP in the study years who had previously had siblings with some degree of TP. Two were known NAIT cases and a third was almost certainly NAIT (see note for Case#4). One was a stillborn (Case #12 linked to #14 in Table 1). The fifth case (#2) with severe TP was born outside the timeframe of the cord blood audit, but had a previously affected sibling with mild TP. The mother (Fijian Indian) had ITP.

The remaining 13 cases of moderate or mild TP were often associated with grand multiparity, diabetes or ITP.

Owing to low numbers of cases we did not perform formal statistical testing.

## 4 Discussion

From over 60000 routine cord blood collections, TP was noted in 1% and severe TP in 0.025% (2.5/10000). NAIT was suspected or proven in 0.013%. Only 2/62082 (0.003%) were from neonates whose mothers identified as Maori or Pacific Island families. Even in these cases, the diagnosis was putative as there were no repeat neonatal follow up platelet counts or immunological studies. Siblings of the Maori infant had normal counts suggesting a cause other than NAIT or that the platelet antigens were different in the other siblings. The infant born to the Tongan woman had no other siblings.

The overall percentage of cases with any TP in our study was within the range of other reports, whilst cases of severe TP and NAIT were considerably lower (Table 3) Most of the comparator studies were carried out in Europe (and one in Canada).

That percentages of severe TP and, in particular severe NAIT, were lower in our cohort, likely reflected the different ethnic makeup and frequencies of different platelet antigens [16]. The main ethnicities at Middlemore Hospital maternity were approximately 20% Maori, 30% Pacific Island and 26% European [17]. The prevalence of severe TP which may have been due to NAIT (0.008% for Maori, 0.005% for Pacific Island) was very low and even this may be an over‐estimate in view of the unconfirmed diagnosis. The most common cause of NAIT in approximately 80% of European infants is anti‐HPA—1afollowed by anti‐HPA—5b^10^, whilst a higher frequency of anti—HPA—15 antibodies was detected in a study involving Polynesian and Maori people [16]. In Japanese and other Asian populations anti‐HPA—4b and anti‐HPA—6b have been described, and antibodies to other HPAs such as HPA—2, —3 and—15 have also been implicated in NAIT [10, 16, 18, 19].

In our study, classic severe NAIT with severe TP and antibodies to HPA—1 was present in several European infants and the diagnosis suspected or proven in 6 cases overall (0.036% of European descent). Only two cases of NAIT received any treatment for TP and there were no records of severe haemorrhage (with one suspected case of NAIT having a petechial skin rash). However, there is a severe risk of spontaneous bleeding with counts below 5 × 10^9^/L^1^ and such bleeding, although rare, may take the form of intracranial haemorrhage with adverse neurological outcome or death [8, 12, 15, 20].

There may be significant implications in subsequent pregnancies for families affected by NAIT as the risk of recurrence is high [21]. Although TP was detected in the siblings of thrombocytopenic infants in this study, severe TP was uncommon. Severe NAIT or maternal ITP were the main causes of low counts in siblings. In the remaining cases (mainly mild TP), the women were often multiparous or had diabetes. TP has been described in infants of diabetic mothers, although the reasons are unclear [22]. In addition, 30% of multiparous women may be immunised against HLA class 1 (also a platelet antigen) [23]. Anti‐HPA and HLA antibodies increase with increasing parity [24] and 20% of women delivering at Middlemore Hospital were in their third or higher pregnancy [25].

Strengths of this study were the large numbers of unselected births. The population of women was diverse and predominantly non‐European, enabling us to estimate the potential for severe NAIT amongst well newborns of Maori and Pacific Island peoples, whose platelet antigens are likely to be different from those of classic NAIT [16].

As we were able to review platelet counts in siblings of a high proportion of affected asymptomatic term neonates we could determine that the risk of finding severe TP in other siblings was very low.

Weaknesses were that detailed testing for platelet antigens and antibodies was not carried out in many of the cases. By following up siblings of asymptomatic infants we found minimal clinical effect of this. It was possible that there may have been more than one cause of the TP (especially in symptomatic infants) and conversely in several cases the initial count may have been spuriously low and was not repeated. We did not include preterm infants and not all stillbirths had a post‐mortem. We did not account for early pregnancy loss due to NAIT and did not routinely review maternal platelet counts. The south Auckland demographic may not reflect the wider make up of all Maori and Pacific Island peoples.

We conclude that severe TP potentially due to NAIT was very rare in infants of Maori and Pacific Island ethnicity. Routine cord blood platelet counts in asymptomatic term infants in this hospital population were not of great value for detecting severe NAIT and we do not advocate for routine cord blood platelet counts. Our results provide impetus for wider information gathering about NAIT amongst Maori and Pacific people. In this regard, establishing a national registry of NAIT cases would be extremely useful and, as the national blood service performs centralised testing for NAIT, this is feasible. Registry cases should also include stillbirths and those that come to the attention of fetomaternal units.

## Conflicts of Interest

The authors declare no conflicts of interest.

## Data Availability

Original raw data will not be available.
